# Chest Examination 3.0 With Wireless Technology in a Clinical Case Based on Literature Review

**DOI:** 10.7759/cureus.39464

**Published:** 2023-05-25

**Authors:** Marco Umberto Scaramozzino, Guido Levi, Giovanni Sapone, Ubaldo Romeo Plastina

**Affiliations:** 1 Department of Pulmonology, La Madonnina Clinic, Reggio Calabria, ITA; 2 Department of Thoracic Endoscopy, Tirrenia Hospital, Reggio Calabria, ITA; 3 Department of Pulmonology, ASST Spedali Civili, Brescia, ITA; 4 Department of Clinical and Experimental Sciences, University of Brescia, Brescia, ITA; 5 Department of Cardiology, Policlinico Madonna della Consolazione, Reggio Calabria, ITA; 6 Department of Radiology, ECORAD Study of Radiology and Ultrasound, Reggio Calabria, ITA

**Keywords:** crackles, chest ct, obstructive and restrictive lung diseases, thoracic objective examination 3.0, artificial intelligence (ai)

## Abstract

Physicians use auscultation as a standard method of thoracic examination: it is simple, reliable, non-invasive, and widely accepted. Artificial intelligence (AI) is the new frontier of thoracic examination as it makes it possible to integrate all available data (clinical, instrumental, laboratory, functional), allowing for objective assessments, precise diagnoses, and even the phenotypical characterization of lung diseases. Increasing the sensitivity and specificity of examinations helps provide tailored diagnostic and therapeutic indications, which also take into account the patient's clinical history and comorbidities.

Several clinical studies, mainly conducted in children, have shown a good concordance between traditional and AI-assisted auscultation in detecting fibrotic diseases. On the other hand, the use of AI for the diagnosis of obstructive pulmonary disease is still debated as it gave inconsistent results when detecting certain types of lung noises, such as wet and dry crackles. Therefore, the application of AI in clinical practice needs further investigation. In particular, the pilot case report aims to address the use of this technology in restrictive lung disease, which in this specific case is pulmonary sarcoidosis.

In the case we present, data integration allowed us to make the right diagnosis, avoid invasive procedures, and reduce the costs for the national health system; we show that integrating technologies can improve the diagnosis of restrictive lung disease. Randomized controlled trials will be needed to confirm the conclusions of this preliminary work.

## Introduction

Lung sounds have been used to understand the health of the respiratory system since ancient times. The introduction of Laennec’s stethoscope in the nineteenth century set a milestone in the diagnosis of lung diseases; nowadays, we are witnessing a revolution in respiratory assessment thanks to the introduction of more sensitive and specific methods and computerized spirometry [1,2]. In the last decade, we have improved our understanding of lung sounds, even though we still lack a comprehensive model of thoracic and pulmonary acoustics.

Traditional stethoscopes have limitations, especially in detecting the crackles that characterize restrictive respiratory diseases. Today, mobile applications can help physicians by recording, storing, playing, and analyzing respiratory sounds, complementing the chest examination. Simulation studies have shown that mobile apps have high accuracy, sensitivity, and specificity in the detection of fine crackles, while they do not perform as well in detecting coarse crackles. It has also been shown that a mobile app can help assess lung sounds in patients [3]. Some studies have tried to describe the audiological characteristics of wheezing and crackles in adults and children using digital stethoscopes [4]. It has been shown that digital stethoscopes can analyze and display data in new ways, work in real-time, classify lung sounds following the conventional categories, and differentiate single sounds when multiple sounds are present simultaneously [5]. Electronic stethoscopes offer several advantages over analog stethoscopes as they can reduce background noise, amplify, store, and transmit sounds. However, clinicians may find it difficult to switch to a digital stethoscope because of the different acoustics [6].

Lung auscultation has a key role in the diagnosis of respiratory diseases. Standardized nomenclature and computational analysis of the sounds have improved the technique; however, auscultation lacks reproducibility as it relies on the clinician’s judgment [7]. Artificial intelligence (AI) can overcome this limitation and help diagnose lung diseases. Artificial neural networks (ANN) and K-nearest neighbors are the most used machine learning algorithms for the analysis of lung sounds. Kandasamy et al. showed that ANN classified different breath sounds with 100% and 94.02% accuracy during training and testing, respectively, proving that ANN is efficient at processing and classifying complex non-linear data [8]. Another study concluded that AI could detect fine and coarse crackles in respiratory sounds recorded by different digital stethoscopes, although there were some device-dependent differences [9]. Grzywalski et al. integrated the thoracic examination of children with an AI diagnostic algorithm, improving both the sensitivity and specificity of the examination, reaching values close to 100% diagnostic specificity and sensitivity [10]. It will be essential to perform independent validations of the AI tools that enter medical care to ensure quality control [7].

## Case presentation

A 67-year-old Caucasian female presented to the clinic for nocturnal dyspnea and cough, wheezing, and moderate exertional dyspnea (mMRC: 3). She was taking inhaled corticosteroids, a once-daily long-acting beta-2 agonist, a long-acting muscarinic antagonist, and montelukast, but the therapy had not given any clinical benefits. The patient was allergic to non-steroidal anti-inflammatory drugs type II and different antibiotics (cephalosporins, penicillin, sulfonamides, pyramidoids), salicylates, and barbiturates. She reported nasal polyposis, never smoked, had insulin-independent diabetes mellitus (for which she was taking metformin twice daily), obesity (BMI: 31), systemic hypertension, hypercholesterolemia, and hypertriglyceridemia. She underwent coronary stent placement two years before and was on clopidogrel therapy. She had been diagnosed with overlap syndrome, asthma-chronic obstructive pulmonary disease overlap syndrome and atopy, but she had never performed a bronchodilator reversibility test. She showed the results of a sleep cardiorespiratory monitoring that recorded an apnea-hypopnea index (AHI) of 6.7 that, according to the pulmonologist who previously assessed it, was not indicative of obstructive sleep apnea (OSA). Despite the high AHI and the evidence of supine-related OSA (supine AHI/non-supine AHI > 3:1), the patient was not under any treatment.

She presented three previous chest CT scans: the first from 2018 showed a sharp-edged nodule of 1.4 cm in diameter with a sharp-edged nodule of 8 mm at the apical-medial segment of the left lower lobe, a small fibrotic thickening, and a mediastinal lymph node of 3 cm in diameter. The second scan from 2021 showed nodular formations of 2-3 mm in the apical segment of the left lower lobe and parenchymal thickening with air bronchogram in the medial segment of the middle lobe; there were thickenings at the base of the lungs and a 3 cm hilar lymphadenopathy. The third scan from February 2023 showed calcified centrilobular nodules and calcified hilar lymphadenopathy with areas of air trapping and bilateral bronchiectasis.

Table 1 compares the results of the spirometry tests the patient carried out in September 2022 and February 2023.

**Table 1:** Comparison between the first spirometry from September 2022 and the complete spirometry from February 2023. In 2023, the patient still shows signs of an obstructive condition with a further reduction of the lung volumes and air trapping also due to poor therapeutic adherence FEV1%: percentage of predicted value of FEV1, FVC%: percentage of predicted value of FVC, PEF: peak expiratory flow, FEV1: maximum expiratory volume at first second, FVC: forced vital capacity, TLC: total lung capacity, RV: residual volume, RV/TLC: ratio of total lung capacity to residual volume expressed as a percentage of the predicted value, FEF25-75%: forced expiratory flow between 25% and 75% of FVC

FEV1%	FVC%	FEV1/FVC%	PEF	FEF_25-75 %_	RV	TLC	RV/TLC
71%	86%	89%	61%	35%	134%	88%	145%
66%	79%	82%	67%	39%	124%	93%	135%

Table 2 shows the results of the blood test done in January 2023.

**Table 2:** Laboratory exams carried out in January 2023 mm/h: millimeters per hour, mcg/ml: micrograms/milliliter, mg/dl: milligrams/decilitre, U.I./l: international unit/liter, U.I./mL: international unit/milliliter, mg/24h: milligrams in 24 h, mmol/mol: millimoles/moles, ANA: antinuclear antibodies, ANCA: antineutrophil cytoplasmic antibodies, ENA: extractable nuclear antigen, Anti-CCP: anti-cyclic citrullinated peptides, ACE: angiotensin-converting enzyme, CRP: C-reactive protein, ESR: erythrocyte sedimentation rate, GOT: glutamic-oxaloacetic transaminase, LAC: lupus anticoagulant, GPT: glutamate pyruvate transaminase

Autoimmune	Results	Blood Test	Results
ANA	Negative	GOT	23 U/l
P-ANCA	Negative	Glycemia	214mg/dl
ENA	Negative	Antiphospholipid antibodies	Negative
C-ANCA	Negative	Serum Creatinine	1,01mg/dl
Rheumatoid factor	9 UI/mL	Azotemia	60 mg/dl
Calciuria	290mg/24h	Coagulation	Negative
Anti-CCP	Negative	D-Dimer	Negative
Lysozyme	16 mcg/ml	LAC (lupus anticoagulant)	Negative
ACE	<1 U/l	Glycated hemoglobin	8,8% mmol/mol
CRP	2 mg/dl	Leukocyte count	230 Eosinophils cell
ESR	30 mm/h	GPT	30 U/l

**Figure 1:**
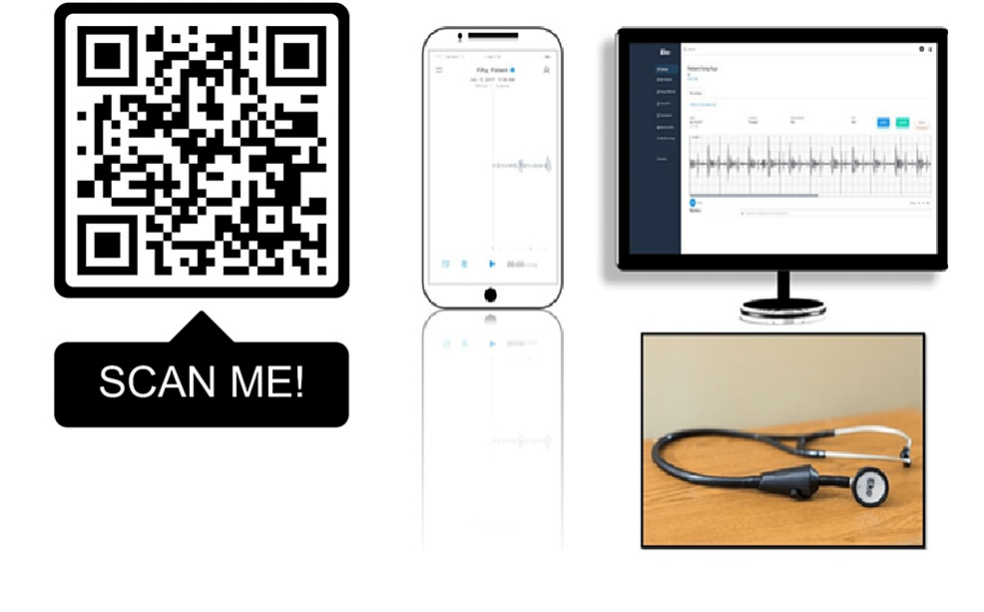
Eko Core system, smartphone interface, web interface and stethoscope, and respective QR code that allows the patient's thoracic examination findings to be heard through earphones. If you scan the QR code in the image, you can hear the objective thoracic examination performed on the patient QR code: two-dimensional barcode

When the patient presented to the clinic, we auscultated the lungs with a digital stethoscope, the Eko Core stethoscope (Figure 1), and detected diffuse velcro crackles over the entire area and signs of airway obstruction on forced expiration (moans and hisses). The lung sounds were recorded, assessed, and compared with the chest CT findings. The EKO mobile application used on the telephone makes it possible to record, store, reproduce, and possibly modify the filter of the sound heard by abolishing background noise and making the auscultation of the thorax in fibrotic pathologies more specific and sensitive. The patient was then diagnosed with bronchial asthma with hypereosinophilia and nasal polyposis associated with bilateral bronchiectasis and reduced lung volumes, stage II pulmonary sarcoidosis (diffuse pulmonary lymphadenopathy and nodules with calcifications) with renal involvement, mild OSA with higher frequency in a supine position associated with obesity, and uncontrolled type 2 diabetes mellitus. Positional therapy was prescribed for the OSA, and the patient was referred for evaluation of diabetes and renal assessment.

## Discussion

Auscultation is a standard method to hear lung sounds, and it is widely used as it is simple, reproducible, and non-invasive. Auscultation can be either direct or indirect: during direct auscultation, the physician listens to the lungs with her ear, whereas during indirect auscultation, she uses a stethoscope. Since the introduction of the acoustic stethoscope, indirect auscultation has gradually replaced the direct method [11].

Discerning normal and abnormal respiratory sounds (such as crackles, wheezes, and hisses) is crucial for an accurate and early diagnosis, which can prevent chronic respiratory diseases. For instance, an early diagnosis of hypersensitivity pneumonitis prevents pulmonary fibrosis, reversing a poor prognosis.

Lung sounds give valuable information about the physiological and pathological condition of the lungs and airways. Indeed, the first step of non-invasive diagnoses of respiratory diseases involves auscultation and comparison with the medical history. However, the detection of abnormal lung sounds depends on the skills and expertise of the physician. To overcome these limitations, different digital methods have been proposed. Electronic stethoscopes automate the detection of lung sounds by processing the signal with time-frequency analysis or time-varying autoregressive modeling [12].

A Korean clinical study tested the accuracy of deep learning convolutional neural networks for the analysis of lung sounds. The predictive model accurately detected abnormal sounds and was also able to classify them into three categories, namely, crackles, wheezes, and rhonchi. The model proved to be more precise than human evaluation [13]. Similar results were obtained in a pediatric study where abnormal sounds were categorized into crackles, rattles, and buzzes with such a high accuracy that it was considered suitable for the initial screenings and follow-ups of patients with respiratory diseases [14].

In another pediatric clinical study, breath sound recordings were collected in a clinical setting that was full of other baby noises, cries, voices, and movements. The AI algorithm analyzed 93.3% of the recordings, reaching an accuracy comparable to one of the experienced pediatric pulmonologists [15]. Cardiologists compared the results of personal heart auscultation and echocardiogram with the recordings of an EKO Core stethoscope and found that the categorization was comparable and showed moderate reliability [16]. Another study demonstrated how lung auscultation with a new-generation wireless stethoscope is possible in hospitalized patients with SARS-CoV-2 pneumonia and allows the assessment of velcro crackles, which indicate a poor prognosis if widespread and audible [17]. Finally, Horimasu et al. compared how a machine-learning-based algorithm they developed and X-rays performed in the diagnosis of interstitial lung diseases (ILDs). The researchers concluded that quantifying fine crackles using an electronic stethoscope and their algorithm was more sensitive than X-rays in determining the presence of ILDs. Their study demonstrated that the quantification of fine crackles can predict high-resolution computed tomography results and help the diagnosis of ILDs [18].

In the clinical case we present, our approach, which integrated data from function tests, instruments, and AI, allowed us to make the right diagnosis. Our diagnosis was complicated because, despite the radiological evidence of calcified central-lobular nodules and mediastinal lymph nodes, the laboratory tests and the spirometry results initially suggested a different diagnosis by showing low angiotensin-converting enzyme levels and the absence of a restrictive ventilatory defect. The experiences with this instrument in the pathology of sarcoidosis is a clinical diagnosis based essentially on the integration of data: spirometric, radiological, and laboratory, and represent an objective examination 3.0 that allows a precision diagnosis with increased diagnostic sensitivity and specificity.

## Conclusions

Researchers have started to introduce AI in thoracic examinations, but there are still doubts about its use in the diagnosis of obstructive pulmonary diseases, while different clinical trials have already confirmed its high potential in the detection of fibrotic diseases. Mobile apps integrated with a digital stethoscope can improve the sensitivity and specificity of the thoracic examination, and machine learning can generate algorithms that improve the diagnostic efficiency of lung diseases. I think that the interpretation of the integrated data is essential to make a better and more precise diagnosis, and technology has made and will make further advances in recent years that it will be up to us pulmonary physicians to grasp to try to make correct diagnostic and therapeutic paths in patients with fibrotic lung diseases.

The real strength of the objective thoracic examination is the integration of all available data (clinical, instrumental, laboratory, functional) to achieve precision in the diagnosis and to characterize the phenotype of lung diseases. Electronic auscultation allows for storing pathological and physiological lung sounds that can be then compared to other types of examinations, such as chest high-resolution CT scans. In this clinical case, the use of an electronic stethoscope allowed us to diagnose an interstitial lung disease with percentages of specificity and sensitivity that are in line with the ones reported in the literature (80% and 90%, respectively).
